# Personal

**Published:** 1909-01

**Authors:** 


### PERSONAL.

---

Dr. Edward N. Brush, formerly of Buffalo, and one time editor of this Journal, now superintendent of the Sheppard and Enoch Pratt Hospital, Baltimore, was tendered a dinner in the latter city, December 11, 1908. The purpose of the participants was to express their personal regard for Dr. Brush, and their appreciation of the great service he has rendered in the care of the insane, during the past thirty years. The dinner was attended by nearly a hundred physicians and friends and was a conspicuous success.

---

Dr. A. L. Benedict, of Buffalo, whose specialty is diseases of the digestive organs, has removed to the Hotel Raleigh, 354 Franklin Street. Hours, until one, and by appointment. Telephones: Bell, Tupper 320. Frontier, 720—10193.

---

Dr. Arthur G. Bennett, of Buffalo, and not Dr. Alice Bennett as the Journal announced in its November issue, has been appointed to the attending staff of the German Deaconess's Hospital. The error is to be regretted, and we attempt to make amends by this announcement, even though it is somewhat belated.

---

Dr. DeWitt G. Wilcox, formerly of Buffalo, where he resided for 22 years, has removed to Boston, which becomes his residence for the future. He will be associated with Dr. N. W. Emerson, in the conduct of the Emerson Hospital, and also becomes associate in the chair of gynecology at the Boston University. The many friends of Dr. Wilcox in Buffalo and Western New York, will deeply regret his departure.

---

Dr. Renwick R. Ross, who for sixteen years has been superintendent of Buffalo General Hospital, recently received an invitation to take similar charge of Bellevue and allied hospitals at

New York. After considering the matter with its advance in salary, Dr. Ross declined the appointment, prefering his present field of duty.

